# Circulating Biomarkers for Prediction of Objective Response to Chemotherapy in Pancreatic Cancer Patients

**DOI:** 10.3390/cancers11010093

**Published:** 2019-01-15

**Authors:** Fleur van der Sijde, Eveline E. Vietsch, Dana A. M. Mustafa, Marc G. Besselink, Bas Groot Koerkamp, Casper H. J. van Eijck

**Affiliations:** 1 Department of Surgery, Erasmus MC, University Medical Center Rotterdam, 3000 CA Rotterdam, The Netherlands; f.vandersijde@erasmusmc.nl (F.v.d.S.); e.vietsch@erasmusmc.nl (E.E.V.); b.grootkoerkamp@erasmusmc.nl (B.G.K.); 2 Department of Pathology, Erasmus MC, University Medical Center Rotterdam, 3000 CA Rotterdam, The Netherlands; d.mustafa@erasmusmc.nl; 3 Department of Surgery, Cancer Center Amsterdam, Amsterdam UMC, University of Amsterdam, 1105 AZ Amsterdam, The Netherlands; m.g.besselink@amc.uva.nl

**Keywords:** pancreatic cancer, chemotherapy response, FOLFIRINOX, gemcitabine, predictive circulating biomarker

## Abstract

Pancreatic cancer is a lethal disease with increasing incidence. Most patients present with advanced disease, for which palliative systemic chemotherapy is the only therapeutic option. Despite improved median survival rates with FOLFIRINOX or gemcitabine chemotherapy compared to the best supportive care, many individual patients may not benefit from chemotherapy. Biomarkers are needed to predict who will benefit from chemotherapy and to monitor a patient’s response to chemotherapy. This review summarizes current research and future perspectives on circulating biomarkers for systemic chemotherapy response.

## 1. Introduction

Pancreatic ductal adenocarcinoma (PDAC) is the twelfth most common cancer worldwide. Its incidence is rising; PDAC will likely be the second leading cause of cancer-related deaths in 2030, exceeding the mortality from colorectal and breast cancer [1]. The mortality rate of PDAC is almost equal to the incidence rate, with a 5-year survival rate of only 7.7% [2]. Approximately 80% of patients are diagnosed with advanced disease and are therefore not eligible for tumor resection [3]. For those patients, chemotherapy is currently the standard treatment, while more effective therapies are lacking. Patients with resectable PDAC are increasingly offered neoadjuvant treatment instead of upfront surgery followed by adjuvant systemic treatment. Pre-operative chemotherapy is likely to be the future standard [4,5,6,7,8,9,10,11]. 

Different chemotherapy regimens are offered, but FOLFIRINOX (a combination of fluorouracil, leucovorin, irinotecan and oxaliplatin) is currently the treatment of choice for patients with metastatic disease and locally advanced pancreatic cancer (LAPC). For metastatic PDAC, FOLFIRINOX is superior to gemcitabine in terms of patient survival benefit [12,13,14]. The median overall survival (OS) of metastatic PDAC patients is 11.1 months when treated with FOLFIRINOX, compared to 6.8 months when treated with gemcitabine [12]. A recent meta-analysis has shown that this holds as well for LAPC. The median OS in patients treated with FOLFIRINOX was 24.2 months [13] versus 6–13 months in patients treated with gemcitabine [15,16]. Because of its lower toxicity profile, gemcitabine is still often used, particularly in patients with a poor performance status [12] and as adjuvant treatment after surgical resection. Only few articles have been published on adjuvant FOLFIRINOX treatment. A recent randomized controlled trial showed a median OS of 54.4 months in the FOLFIRINOX group versus 34.8 months in the gemcitabine group [17]. It should be mentioned that adjuvant gemcitabine combined with capecitabine might be more effective than gemcitabine monotherapy. The ESPAC-4 trial showed a median overall survival of 28.0 months for gemcitabine plus capecitabine versus 25.5 months for gemcitabine monotherapy [18].

In a multicenter, randomized, phase 2–3 trial in metastatic patients, FOLFIRINOX treatment was found associated with higher response rates than gemcitabine, based on the RECIST criteria for radiologic monitoring of the disease [12,19]. Nevertheless, a recent meta-analysis still showed a response rate of only 25–30% in metastatic patients [20]. This could mean that the large majority of patients will suffer from side effects without benefiting from FOLFIRINOX treatment. Toxicity is a serious problem in chemotherapy treatment. Especially with the FOLFIRINOX combination regimen, grade 3 and 4 toxicity rates are as high as 60–70% [12,13,20,21,22]. For gemcitabine monotherapy, toxicity rates are significantly lower [12,23,24]. Identifying non-responders is crucial to avoid exposing patients to the adverse events and incurring costs of ineffective treatment. 

Currently, response to chemotherapy is evaluated with the use of computed tomography (CT) scans. The RECIST criteria classify response in to four classes. Complete response (CR) is defined as the disappearance of all target lesions; partial response (PR) as a decrease of >30% in the sum of diameters of target lesions; and progressive disease (PD) is defined as an increase of >20% in the sum of diameters of target lesions or the appearance of a new cancer lesion. Stable disease (SD) shows neither sufficient shrinkage nor increase to be classified as PR or PD [19]. In case of (partial) response or SD, treatment will be continued, while PD is a reason for treatment discontinuation. Although the RECIST criteria have been validated for multiple solid cancers and show a good inter- and intra-observer agreement [25,26,27], the value of radiologic response assessment for PDAC is still being debated. Measurements of the primary tumor are difficult and less reliable in view of its invasive growth pattern and fibrotic aspects combined with inflammation [28,29]. Moreover, treatment response is not only reflected by a decrease in tumor size. Therefore, predictive biomarkers for chemotherapy response, and for FOLFIRINOX in particular, in PDAC patients would be of great value to spare patients this toxic treatment.

A biomarker is a measurable characteristic that can be used as an indicator of physiologic and pathologic processes. Two main categories are distinguished: prognostic and predictive biomarkers. Prognostic biomarkers provide insight into patient outcome, independent of the received treatment. These biomarkers can give an estimation of survival, time to disease progression or type of progression. Predictive biomarkers, on the other hand, can identify subsets of patients who would benefit from specific cancer treatment, and thus can guide treatment decisions. A biomarker can only be termed predictive if its predictive value was confirmed in a study with at least two treatment comparison groups, preferably a randomized controlled trial [30]. An ideal biomarker is cheap to obtain, easy to analyze, non-invasive for the patient, and can be collected repeatedly. 

Circulating biomarkers are gaining interest in cancer research due to the many advantages over tumor tissue biomarkers. For PDAC as well as other cancers, liquid (blood) biopsies are preferred in the search of predictive and prognostic biomarkers, because the procedure is less hazardous than percutaneous or endoscopic tumor biopsies. Not all PDAC patients undergo a tumor biopsy and the primary tumor is resected in only a minority of patients. Moreover, the representation of intra-tumoral heterogeneity in a single tumor biopsy is limited, which can possibly be overcome by using liquid biopsies. These represent a more complete make-up of the primary tumor and/or metastatic sites [31]. Blood samples, e.g., plasma or serum, are a great source of cell-free DNA, RNA, exosomes, proteins and metabolites. Repeated measurements in peripheral blood are easy to obtain and preferred over multiple tumor biopsies [32]. 

In this review, we will discuss current research and future perspectives on circulating biomarkers predictive for chemotherapy response used for pancreatic cancer patient stratification.

## 2. Results

A total of 27 articles met the inclusion criteria and will be discussed in this review.

### 2.1. Conventional Tumor Markers

Carbohydrate antigen 19-9 (CA19-9) is a sialylated Lewis blood group antigen associated with different cancers, including PDAC [33]. This marker was first described by Koprowski et al. as a tumor associated antigen in colorectal cancer cell lines [34]. CA19-9 is widely studied as a diagnostic, prognostic and predictive biomarker in PDAC. However, up to 20% of the population is Lewis negative and cannot synthesize CA19-9 [35]. 

Thirteen articles deal with the role of conventional tumor markers as a predictor of chemotherapy response. For an overview, see Table 1. Four of the studies concerned focus on the predictive value of baseline levels of CA19-9 to predict objective response. Posttreatment CA19-9 levels were measured in two studies. Twelve studies investigated the predictive value of changes in CA19-9 levels over time. 

#### 2.1.1. Baseline CA19-9

Boeck et al. investigated baseline CA19-9 levels in 68 patients with LAPC, metastatic, and recurrent disease before receiving gemcitabine- or capecitabine-based treatment. In patients with non-progressive disease (SD+PR+CR), the median baseline level was 341.5 U/mL. In patients with PD after chemotherapy, the median baseline level was 5810.5 U/mL (*p* = 0.006) [36]. Koom et al. measured CA19-9 levels before chemoradiotherapy (gemcitabine or paclitaxel) in 69 patients with borderline resectable PDAC or LAPC. The median level in responders (PR+CR) was 661 U/mL; that in non-responders (PD+SD) was 518 U/mL (*p* = 0.78) [37]. An et al. reported a median CA19-9 level of 682 U/mL in the complete study population, including 61 patients with LAPC and metastatic disease receiving gemcitabine-based treatment. The median CA19-9 level was 682 U/mL. The objective response rate (ORR) in the patients with a baseline level below 682 U/mL was 43.5% versus 15.8% in the patients with a baseline level above 682 U/mL. (*p* = 0.051) [38]. In an article by Yoo et al., 84 LAPC patients underwent chemoradiotherapy with 5-FU or capecitabine. The response rate, including CR and PR, in patients with a baseline CA19-9 level below 100 U/mL was 51.9% versus 37.5% in patients with a baseline CA19-9 level between 101-400 U/mL and 15.2% in patients with a baseline CA19-9 above 400 U/mL (*p* = 0.009) [39]. In conclusion, baseline CA19-9 levels in non-responders are higher than those in responders.

#### 2.1.2. Posttreatment CA19-9

Boeck et al. and Koom et al. investigated the role of posttreatment CA19-9 levels as a predictor of radiologic response. Boeck et al. found different posttreatment levels in patients with PD and patients with non-PD. At the time of re-staging, eight weeks after start of treatment, median CA19-9 levels were 135.0 U/mL in patients with non-PD and 6428.0 U/mL in patients with PD (*p* < 0.001) [36]. In contrast to baseline CA19-9 levels, posttreatment CA19-9 levels showed statistically significant difference between responders and non-responders. Koom et al. reported a median CA19-9 level in responders of 80 U/mL versus 199 U/mL in non-responders (*p* = 0.001) [37]. This means that not only baseline levels, but also posttreatment levels of CA19-9 are higher in patients without response to treatment. 

#### 2.1.3. CA19-9 Changes

Several authors looked into the predictive value of decreasing CA19-9 levels under chemotherapy. Boeck et al. quantified changes in CA19-9 from baseline at different time points. After eight weeks, for example, median CA19-9 levels in patients with non-PD had decreased by 65.2% versus 17.4% in patients with PD (*p* < 0.001) [36]. In the study by Koom et al., the median decrease from baseline in responders was 93% and in non-responders 72% (*p* = 0.002) [37]. In the study by An et al., the ORR in patients with CA19-9 level decrease ≥25% was 47.8% versus 10.5% in the group with <25% decrease [38]. Gogas et al. studied 39 patients with LAPC and metastatic disease, all treated with a combination of 5-FU, cisplatin and epirubicin. A decrease in CA19-9 ≥15% was considered a biochemical response to treatment; an increase ≥15% was considered biochemical progression of disease. CA19-9 decrease showed a sensitivity of 67%, specificity of 69%, positive predictive value (PPV) of 20% and negative predictive value (NPV) of 87% for partial response as based on radiologic findings. CA19-9 increase seemed a slightly better prediction tool for progression with a sensitivity of 86%, specificity of 67%, PPV of 37% and NPV of 90% [40]. Halm and colleagues defined CA19-9 response as a decrease of >20% and quantified CA19-9 levels in 36 patients with LAPC and metastatic disease, treated with gemcitabine. After eight weeks of treatment, four patients achieved partial objective response, as measured on CT scans. All four of these patients showed a CA19-9 decrease >20%, indicating biochemical response. Still, 19 out of 25 patients with SD showed the same biochemical response to treatment, but also two out of seven patients with PD [41]. It is hard to draw a conclusion because *p*-values are not provided. Ali et al. also defined CA19-9 response as a decrease of ≥20% and studied 18 LAPC and metastatic patients who received gemcitabine treatment. Seven patients out of nine with stable disease and only one patient with partial response on CT scan showed a CA19-9 decrease ≥20%. Two out of eight patients with progressive disease showed decrease in CA19-9 over time [42]. *p*-values are not provided. Stemmler et al. chose a cut-off point of 50% decrease for biochemical response to treatment and studied 77 patients with LAPC and metastatic disease, treated with gemcitabine and cisplatin. All of the complete responders on CT imaging also showed biochemical responses. Seventy of the patients with partial response (91%) were biochemical responders. Fifty-four of the patients with progressive disease on CT (70%) did not have a CA19-9 response as defined above. This resulted in a sensitivity of 93.3%, specificity of 53.2%, PPV of 32.5% and NPV of 97.1% [43]. Wong et al. only included patients with metastatic disease. All 75 patients were treated with gemcitabine-based treatment. The cut-off point for biochemical response to treatment was set at 75% decrease. All (13/13) patients with objective response, 15/43 patients with stable disease and only 1/19 patients with progressive disease showed >75% decrease in CA19-9 levels during treatment (*p* < 0.0001) [44]. The largest study investigating tumor marker CA19-9 as a predictor of response is the one by Chiorean et al., who included 454 patients diagnosed with metastatic disease and treated with gemcitabine with or without nab-paclitaxel. In this study, the ORR was low. Forty of the 252 patients treated with gemcitabine + nab-paclitaxel and 13/202 patients treated with gemcitabine alone showed response to treatment (CR+PR). In the combination treatment group, 38/40 responders showed decrease in CA19-9 after eight weeks of treatment. All 13 responders in the monotherapy group also showed a decrease. Hundred and fifty-eight of the 199 patients with SD (79%) in the nab-paclitaxel group had decreasing CA19-9 levels over time versus 133/170 (78%) in the gemcitabine group. No specific results were given for patients with progressive disease [45]. Tsutsumi et al. studied 90 patients with LAPC and metastatic disease, all treated with gemcitabine chemotherapy. CA19-9 levels after one month of treatment were compared to baseline levels. Patients with SD showed a decrease of 12%; patients with partial response a decrease of 68%; and patients with PD showed a median increase of 27% in CA19-9 level [46]. The study by Azzariti et al. is the only study investigating CA19-9 in metastatic patients treated with FOLFIRINOX. In total, 27 patients were included, of whom 21 received FOLFIRINOX and 6 gemcitabine. Pretreatment and posttreatment levels of CA19-9 were compared within PR, SD and PD patient groups. In the PR group, the mean pretreatment CA19-9 level was 831.5 U/mL and the mean posttreatment level 355.75 U/mL (*p* < 0.01). The corresponding figures for the SD group were 521.4 U/mL and 292.7 U/mL (not significant) and for the PD group 2569.67 U/mL and 3384.44 U/mL (*p* < 0.05) [47]. The authors did not provide inter-group comparisons, but a pattern is shown of decreasing CA19-9 levels over time in patients with response and decreasing CA19-9 levels over time in patients with progression. The most recent CA19-9 study was conducted by Robert et al. in 2017 as part of the ACCORD11/PRODIGE4 trial, comparing FOLFIRINOX to gemcitabine chemotherapy in 160 metastatic patients. For the patient group receiving FOLFIRINOX, the ORR at eight weeks of treatment was 44.0% in patients with a CA19-9 decrease of ≥20% versus 22.9% in patients with a decrease of <20%. For the patient group receiving gemcitabine, the ORR was 23.1% in patients with ≥20% decrease and 0% in patients with a <20% decrease [48]. Although the above studies used different definitions of CA19-9 increase and decrease, the overall consensus is that CA19-9 levels decrease over time in responders and do not decrease or even increase in non-responders.

#### 2.1.4. CEA

Only one study by Boeck et al., focused on carcinoembryonic antigen (CEA), another broadly used tumor marker in cancer prognostics [49,50]. Median CEA levels at baseline were statistically significant different between responders and non-responders. Baseline levels were 3.7 ng/mL in patients with SD or PR and 17.1 ng/mL in patients with PD (*p* = 0.008). Measurement after eight weeks showed median CEA levels of 2.6 ng/mL in patients with SD or PR and 18.1 ng/mL in patients with PD (*p* = 0.002). Patients with response to treatment had a median decrease of CEA of 26.3%, while patients with PD showed no decrease of CEA levels at all (*p* = 0.078) [36].

#### 2.1.5. SPAN-1

Tsutsumi et al. did not only measure the tumor marker CA19-9, but also SPAN-1, a high molecular weight glycoprotein. SPAN-1 is expressed by many pancreatic cancers [51]. This tumor marker has already been investigated as a diagnostic and prognostic tool for PDAC. It is little used however, because its sensitivity and specificity are no higher than those of existing tumor markers such as CA19-9 [51,52]. In the study by Tsutsumi et al., a SPAN-1 change pattern was found similar to that of CA19-9. The median SPAN-1 level decreased by 24% and 48%, respectively, in patients with SD and PR and increased by 11% in patients with PD [46].

### 2.2. Genetic Markers

#### 2.2.1. Single Nucleotide Polymorphisms

The term genetic markers encompass germline single nucleotide polymorphisms (SNPs), somatic cancer mutations, as well as differences in RNA expression. SNPs are variations of a single nucleotide in the DNA and may be associated with development of disease and response to treatment [53]. Theoretically these can all serve as predictors of tumor growth, treatment response or metastasis [32]. An overview of articles describing genetic markers for prediction of chemotherapy is given in Table 2. Two published studies have investigated several SNPs for response to chemotherapy. One, by Dong et al., included 131 resectable patients treated with neoadjuvant gemcitabine based chemotherapy. It focuses on 15 SNPs in genes already known for their roles in DNA mismatch repair (MMR) [54]. MMR is necessary for recognition, removal, and repair of DNA damage generated during DNA replication. Deficiencies in MMR could interfere with the response to chemotherapy, because chemotherapeutic stress on the tumor cells results in decreased apoptosis [55]. Five of the 15 SNPs were associated with response to preoperative chemotherapy. Genotype *MSH2* G322D with SNP GG was associated with an ORR of 88.1%; genotype *MSH2* G322D with SNP GA/AA was associated with an ORR of 63.2% (*p* = 0.04). *MSH2* IVS12-6T>C with SNP TT was associated with an ORR of 92.8%, versus 71.9% with SNP TC/CC (*p* < 0.001). Genotype *MSH3* P231P with SNP GG was associated with an ORR of 89.2%, versus 65.4% with the GA/AA genotype (*p* = 0.002). ORR for *TREX1* Ex14-460C>T with SNP TT/CT and CC were 88.5% and 75.6% respectively (*p* = 0.047). *TP73* Ex2+4G>A with SNP GG showed an ORR of 94.3%, SNP GA/AA 72.2% (*p* < 0.001) [54]. The second study, by Tanaka et al., selected 17 SNPs that have been found involved in the metabolism of gemcitabine. 149 LAPC patients were included, who all received gemcitabine-based chemotherapy and radiation. Only two genotypes were associated with objective response to treatment. The ORR for genotype *CDA* A-76C (K27Q) with SNP AA was 72.4% and for SNP AC/CC 51.8% (*p* = 0.017). The ORR for *hENT1* A-201G with SNP AA/AG was 95.0% and for SNP GG only 33.3% (*p* = 0.019) [56]. Obviously, comparison of these two articles is futile because they address different SNPs. Together, however, they make clear that even small genetic alterations can influence a patient’s response to chemotherapy.

#### 2.2.2. Circulating Tumor DNA

Circulating cell-free tumor DNA (ctDNA) is released in the bloodstream as a result of apoptosis or necrosis of tumor cells and represents the molecular make-up of the cancer cells. Blood sampling is much less invasive than tumor biopsies and can represent cancer heterogeneity to a larger extent, compared to a single section from the primary tumor [31]. ctDNA blood levels are higher in patients with larger tumors and tumors that are well vascularized due to increased shedding into the circulation [57]. Theoretically, in case of chemotherapy response, therapy-induced tumor cell death should lead to an increase in ctDNA levels. In practice, however, ctDNA levels will then eventually become undetectable as eliminated cancer cells are no longer shedding their DNA. In the long term, increasing ctDNA levels could indicate disease progression as a result of increasing tumor load. The exact mechanism and timing of ctDNA shedding during and after anti-cancer treatment is not well understood. Moreover, the ctDNA dynamics may be different for drugs with different mechanisms of action. On the other hand, ctDNA can also be used to investigate specific tumor mutations that could be associated with treatment response and prognosis. Most studies that analyzed mutant ctDNA levels were conducted in a prognostic setting. For example, the presence of ctDNA in plasma before treatment was found associated with worse OS in PDAC patients [58,59]. Cheng et al. investigated the ctDNA mutation levels of different genes, including *KRAS*, which has been identified as one of the key players in pancreatic tumorigenesis [60]. Mutational levels were measured at baseline and every eight weeks during chemotherapy with gemcitabine and nab-paclitaxel in 13 metastatic patients. Serial plasma samples were monitored for several mutations, including *KRAS*, *BRCA2* and *EGFR*. Ten of these 13 patients had detectable ctDNA mutations in various genes. While decreasing levels or undetectable levels of ctDNA in the blood over time were associated with objective response, increasing levels were associated with progressive disease. ctDNA levels even increased before progression, visible on a CT scan. Of those 10 patients, eight gained new ctDNA mutations during treatment [61]. Perets et al. investigated ctDNA patterns in 17 patients with metastatic PDAC treated with chemotherapy, details of which were not provided. *KRAS* ctDNA levels during and after treatment increase over time in case of disease progression, as evaluated on radiological imaging. Also in this study, ctDNA levels tended to increase even before progression was visible on CT scans [62]. A study by Del Re et al. investigated ctDNA levels in 13 patients receiving FOLFIRINOX as well as 14 patients receiving gemcitabine with nab-paclitaxel with LAPC or metastatic disease. A decrease in the amount of mutant *KRAS* ctDNA during or after the first cycle of chemotherapy showed a trend towards a better disease control rate (CR+PR+SD) as shown on the first radiologic evaluation scan. The difference in decrease of mutant KRAS ctDNA between responders and non-responders was not statistically significant (*p* = 0.059) [63]. In a pilot study by Tjensvoll et al., the level of mutant *KRAS* ctDNA prior to and during chemotherapy treatment was also significantly higher in patients that developed PD compared to those with SD. Here, too, ctDNA levels increased before disease progression was visible on radiologic imaging [64]. The 14 patients with LAPC or metastatic disease in this study received either gemcitabine or FOLFIRINOX, but with regard to the ctDNA findings, the authors do not distinguish between the two treatments. In none of the aforementioned studies, standardized cut-off values for a decrease or increase in ctDNA levels were determined. From these articles, we can conclude that detectable or increasing ctDNA levels are associated with progressive disease. ctDNA analysis is a promising method for anti-cancer treatment monitoring - even though application in clinical practice is hindered by the lack of established reference values for ctDNA detection levels and fluctuations and the lack of measurement technique standardization.

#### 2.2.3. Long Non-Coding RNAs

Long non-coding RNAs (lncRNA) are RNAs that do not code for proteins [65]. Still, they are important regulators of gene expression and thought to be associated with cancer, cancer recurrence or progression, metastasis, and prognosis [66]. In a recently published study by Wang et al., several lncRNAs were investigated in peripheral blood samples from 62 patients with LAPC or metastatic disease, treated with gemcitabine based treatment. Baseline expression of lncRNAs, preselected based on their association with PDAC according to literature, was measured using real-time polymerase chain reaction (qPCR). The expression levels of three of the 14 lncRNAs investigated showed promise for the prediction of response to treatment. The response rate (CR+PR) in patients with low expression of the lncRNA *PVT1* was 37.1% versus 14.8% in patients with a high expression (*p* < 0.001). The corresponding figures for *HOTTIP* were 37.9% and 18.2% (*p* < 0.001), and those for *MALAT1* 41.1% and 10.7% (*p* = 0.007) [67]. These three lncRNAs have previously been described as prognostic markers in pancreatic cancer. *HOTTIP* for example was found to be upregulated in PDAC tissues. *HOTTIP* silencing can result in proliferation arrest and decrease cell invasion. When *HOTTIP* is inhibited, antitumor effects of gemcitabine are enhanced [66]. *PVT1* has been identified as a regulator of gemcitabine sensitivity, in that overexpression of *PVT1* results in decreased sensitivity [68]. *MALAT1*, too, has been described as a poor prognostic factor in PDAC patients [69]. *MALAT1* promotes tumor proliferation and metastasis through activation of autophagy [70].

### 2.3. Immunologic Markers

Many cancers, including PDAC, may arise from chronic inflammation. Systemic inflammation is often observed in patients with cancer, as a result of the antitumoral response of the host in an attempt to induce tumor destruction. Systemic inflammation can also be tumor-induced, however, and in turn lead to neoplastic progression caused by tumor-promoting effects of immune cells [71]. See Table 3 for an overview of articles investigating predictive immunologic biomarkers for response.

#### 2.3.1. Systemic Inflammation Ratios

Systemic inflammation ratios, such as the neutrophil-to-lymphocyte ratio (NLR), platelet-to-lymphocyte ratio (PLR) and systemic immune inflammation index (SII), reflect the antitumor inflammation capacity of the host and are of prognostic value in PDAC patients [72,73,74]. One study, by Gao et al., investigated the role of the NLR and PLR as a predictive marker in 122 LAPC and metastatic patients, most of whom received gemcitabine treatment. The mean baseline NLR level was 3.81, and the mean PLR level was 142.14. Patients were separated in groups with a low NLR or PLR, or with a high NLR or PLR, using the mean values as cutoff. Disease control (PR+SD) after treatment was accomplished in 47/60 patients (78%) with low NLR levels and in 29/62 patients (47%) with high NLR levels (*p* < 0.001). Disease control was accomplished in 33/60 patients (55%) with a low PLR and in 43/62 patients (69%) with a high PLR (*p* = 0.102) [75]. 

#### 2.3.2. Immune Cells and Cytokines

Regarding the working mechanism of subsets of immune cells instead of absolute numbers of immune cells, regulatory T cells (Tregs) are certainly of interest in immunologic biomarker research in PDAC patients. Tregs are essential in the regulation of immune responses, mostly to maintain tolerance to autoantigens. In cancer, however, Tregs can also suppress antitumor immune response, resulting in an ideal environment for tumor growth, and are associated with poor prognosis [76,77]. Inhibitory cytokines produced by Tregs, such as TGF-β, interleukin-10 (IL-10) and interleukin-35 (IL-35), but also other immune inhibitory cytokines or proinflammatory cytokines produced by other cells and T-cells have been studied as prognostic and predictive biomarkers. 

Two studies have investigated different cytokines as a predictor of response to treatment. The study by Vizio et al. included 58 patients with LAPC or metastatic disease, mainly treated with gemcitabine. Pretreatment levels of interleukines (IL) IL-23, IL-17a and TGF-β1 were significantly lower in responders than in non-responders. The median pretreatment level of IL-23 was 4.68 pg/mL in responders versus 33.17 pg/mL in non-responders (*p* = 0.030). The corresponding figures for IL-17a were 0 pg/mL versus 5.65 pg/mL (*p* = 0.040) and 1469.8 pg/mL versus 1912.85 pg/mL for TGF-β1 (*p* = 0.032) [78]. Interleukin-18 (IL-18), a proinflammatory cytokine produced by macrophages, is also associated with treatment response, according to the study by Usul Afsar et al. In 20 patients with metastatic disease, treated with gemcitabine, lower pretreatment IL-18 levels were also associated with response. The median IL-18 level in responders (PR+SD) was 1273.8 pg/mL versus 1942.8 pg/mL in non-responders (*p* = 0.04) [79]. 

Other interesting immunologic cells are CD44-positive cells. CD44 is a cell-surface glycoprotein involved in cell–cell and cell–matrix interactions and is known to be a cancer stem cell marker. It plays not only a role in cell adhesion, presentation of chemokines, and lymphocyte activation, but also in tumor development and metastasis [80]. Although CD44 is expressed in healthy tissue, it is often up-regulated in different cancer types, including PDAC [81,82,83]. One published study, by Palagani et al., has investigated the role of CD44 as a predictor for response to chemotherapy. Only four patients with metastatic PDAC were included, treated with a combination of 5-FU, oxaliplatin, irinotecan and/or avastin. The expression of CD44 almost immediately decreased after chemotherapy (*p* < 0.05). The level of decrease was associated with objective response, with better outcomes for patients with lower CD44 expression after chemotherapy [84]. 

Another cell-surface interaction molecule, CD40L, has been investigated as a predictor of response. CD40L is expressed by T-cells and plays a role in the adaptive immune response [85]. The soluble form, sCD40L, is produced by activated T-cells, but also by proinflammatory and prothrombotic platelets, leading to elevated serum levels in patients with cancer and autoimmune disorders [86]. Azzariti et al. investigated sCD40L levels in addition to CA19-9 levels, as described before in this review. In patients with PR, mean pretreatment values of sCD40L decreased over time from 11,718.05 pg/mL versus 4689.42 pg/mL (*p* < 0.01). In patients with PD, mean pretreatment values increased from 9351.51 pg/mL to 22,282.92 pg/mL (*p* < 0.01) after treatment. Serum sCD40L levels in patients with stable disease had not statistically significantly changed [47].

The high molecular group box 1 protein (HMGB1) is a nuclear protein involved in DNA organizing and transcription. During necrosis or apoptosis, the HMGB1 protein is released from human cells into the blood stream. A massive release of HMGB1, induced by chemotherapy, will stimulate the immune system such that an anti-tumor response is induced, as well as a pro-tumor effect at the long term [87]. The main receptor of HMGB1, the soluble receptor for advanced glycation end products (sRAGE), has a blocking effect on its extracellular pro-inflammatory functions by binding the protein. This regulation mechanism is thought to be a prognostic factor in cancer and treatment response [87,88]. Wittwer et al. studied 68 patients treated with mainly gemcitabine and compared levels of sRAGE between responders (SD+CR+PR) and non-responders before start of treatment, at days 21 and 42 of treatment, and after treatment at time of staging. Both pretreatement levels and day 21 levels were not significantly different between groups. At day 42, sRAGE levels were higher in responders than in non-responders: median 0.94 ng/mL versus 0.75 ng/mL, respectively (*p* = 0.047) [88]. This pattern was still sustained at day 56: median 1.09 ng/mL versus 0.79 ng/mL [89].

## 3. Discussion

PDAC has a poor prognosis because patients generally present at a late stage of disease, and because of the limited treatment options with disappointing response rates. Patient stratification for systemic therapy in PDAC is not only beneficial for individual patients by preventing morbidity and mortality associated with treatment, it also answers the socioeconomic issue of increasing healthcare costs together with the rise in cancer incidence worldwide. Ineffective (chemotherapy) treatment and toxicity-related complications are an expensive pitfall in the management of PDAC and must be improved in the short term. 

Published studies on circulating biomarkers that can predict chemotherapy response in PDAC patients are scarce. In addition, the previously described studies are difficult to compare because of the different biomarkers investigated and variation in cut-off values (e.g., for CA19-9), study populations (LAPC and/or metastatic patients), and chemotherapy regimens. Therefore, it is hard to accept any of the studied molecules as a predictive circulating biomarker. Moreover, most of the studies lack validation of the investigated biomarkers, and the overall patient populations are small.

None of the studied circulating biomarkers mentioned in this review met the strict criteria for predictive biomarkers. No comparisons are made between a treatment group and a control group, or two different treatment groups, to determine proportions of biomarker-positive and biomarker-negative patients. Thus, the studied biomarkers cannot officially be termed as predictive. However, we believe that these studies provide future directions for further research.

Studies investigating biomarkers specifically for response to FOLFIRINOX or gemcitabine chemotherapy are rare, despite the fact that FOLFIRINOX is currently the best treatment option for patients with locally advanced and metastatic PDAC. Four studies investigated circulating biomarkers in patients treated with FOLFIRINOX [47,48,63,64] and six in patients with fluorouracil (5-FU) [38,39,40,75,78,84] and data for these patients are combined with data from patients treated with other chemotherapeutics, like gemcitabine or nab-paclitaxel. Since FOLFIRINOX is increasingly investigated and implemented as a neoadjuvant therapy in (borderline) resectable patients, the number of patients receiving this therapy will increase, while the short-term response rate will most probably remain similar. Therefore, given the rise in application of FOLFIRINOX, stratifying patients who will benefit from this toxic treatment will be even more important in the future.

An increasing number of ongoing clinical trials are investigating potential predictive biomarkers. For example, in the ongoing Dutch iKnowIT study, blood samples are collected before and during FOLFIRINOX treatment aiming to investigate the predictive value of several biomarkers (i.e., ctDNA and miRNA) to guide FOLFIRINOX therapy. In another randomized controlled clinical trial in the Netherlands (PREOPANC-2) that compares the benefit of neoadjuvant FOLFIRINOX treatment to that of neoadjuvant gemcitabine-based chemoradiation in resectable PDAC patients, blood samples are collected at multiple time points: baseline, during treatment and follow-up, to investigate the predictive value of circulating biomarkers. Consulting (www.clinicaltrials.gov) confirmed that there are several other clinical trials that include liquid biopsies to their protocol. The registered trials including the investigated biomarkers are summarized in Table 4. This is not a complete overview, since many trials are not registered and study protocols are often only known after publication. 

Although this review focuses on circulating biomarkers, tumor biopsies continue to be investigated as a potential source of predictive biomarkers. Histology of tumor biopsies remains pivotal in diagnosis and tumor-specific treatment decision making. In the ongoing HALO-trial for metastasized PDAC patients, the level of hyaluronic acid (HA) in tumor samples is evaluated. Patients with high-HA tumors are randomized to the combination of gemcitabine and nab-paclitaxel only or to gemcitabine and nab-paclitaxel with an addition of PEGPH20, an enzyme that breaks down the hyaluron [90]. In the PRIMUS-001 trial, part of the Precision Panc study, genetic changes in tumor biopsies measured with Next Generation Sequencing are used to predict the efficacy of either FOLFIRINOX combined with nab-paclitaxel or gemcitabine with nab-paclitaxel [91]. Other examples are the two trials conducted by the Seoul National University Hospital and AHS Cancer Control Alberta at which the expression of hENT1 is determined in the resected tumor material in order to decide on adjuvant gemcitabine or 5-FU (Table 4). These concepts, using specific (genetic) changes in pancreatic tumors for tumor-specific treatment decision making, should be encouraged in other clinical trials. 

The majority of the studies focused on CA19-9 as a prognostic and predictive biomarker [36,37,38,39,40,41,42,43,44,45,46,47,48]. The studies showed that lower pre- and posttreatment levels and decreasing levels of CA19-9 over time are associated with chemotherapy response. However, these studies presented various cut-off values and various time points of measurement. Additionally, the patient groups and the chemotherapy regimens vary among the studies, making it difficult to accept CA19-9 as a precise biomarker at this moment. Therefore, we suggest performing a patient level meta-analysis on CA19-9 to identify the best cut-off value that will facilitate using CA19-9 as a treatment predictive biomarker. This requires a large collaborative effort but may be justified because CA19-9 is currently the most commonly used biomarker in clinical practice. In addition, we think that there are other cost effective markers that are easy to obtain and standardize, such as CEA, NLR, PLR, and SII that could be used to predict treatment response. Currently these markers are suggested to be used to monitor PDAC patients in some medical centers. The rapidly evolving –omics techniques, e.g., proteomics, transcriptomics and genomics, empower discovering precise circulating biomarkers. For that reason, we think that all ongoing and future clinical trials will benefit from collection of not only representative tumor biopsies, but also liquid biopsies at multiple time points before, during, and after treatment, in addition to the clinical data. However, it is improbable that a single marker will be sensitive and specific enough to be used as a solitary biomarker. A combination of various molecules and indicators is more likely to predict patient outcome and treatment effect. 

## 4. Materials and Methods

In August 2018, Embase, PubMed, Medline, Web of Science, Cochrane Library, and Google Scholar were searched using the search terms “pancreatic neoplasms”, “biomarkers”, “genetic markers”, “chemotherapy”, “chemoradiotherapy”, “treatment outcome”, and “treatment response” without year restrictions. 

Eligible studies were those using liquid biopsies to investigate predictive biomarkers for response in PDAC patients. Studies that only investigated biomarkers in tissue or did not report the source of biomarkers were excluded, as well as prognostic biomarker studies and biomarker studies on chronic pancreatitis or neuroendocrine tumors of the pancreas. Studies had to report objective response to chemotherapy according to the RECIST criteria. This review article focuses on FOLFIRINOX and gemcitabine treatment, because the majority of PDAC patients receive either one of these therapies. For that reason, only studies that included at least one patient treated with FOLFIRINOX or gemcitabine chemotherapy were included. Letters, editorials, expert opinions, case reports, and non-English language studies were excluded. Eligible studies and those for which information in the abstract was not sufficient for exclusion were read in full. Bibliographies of included publications were searched for other studies.

## 5. Conclusions

Patient stratification is essential for improving the quality of life for patients, and for decreasing unnecessary healthcare costs. Currently, predictive biomarkers for chemotherapy response in PDAC patients are not available. However, various potential predictive biomarkers have been investigated, and some results are promising. Ongoing and future clinical trials will benefit from structured tumor biopsies and blood sampling to investigate potential predictive biomarkers. An increasing number of clinical trials already include liquid biopsies in their study protocol, in addition to tumor biopsies. Combining available clinical data to perform a meta-analysis is required for proper evaluation of existing data on promising biomarkers. It is plausible that a combination of various circulating markers is required to predict treatment response. Identification of the right circulating biomarkers in the near future is crucial for patient therapy improvement. 

## Figures and Tables

**Table 1:** Overview of studies including conventional tumor markers as predictive biomarkers for chemotherapy response in pancreatic cancer patients.

Author	Year of Publication	Number of Patients	Stage of Disease	Treatment	Biomarker
Gogas [40]	1998	39	LAPC (*n* = 21) + metastatic (*n* = 18)	5-FU + cisplatin + epirubicine	CA19-9
Halm [41]	2000	36	LAPC + metastatic	Gemcitabine	CA19-9
Stemmler [43]	2003	77	LAPC + metastatic	Gemcitabine + cisplatin	CA19-9
Ali [42]	2007	18	LAPC + metastatic	Gemcitabine	CA19-9
Wong [44]	2008	75	Metastatic	Gemcitabine + cisplatin (*n* = 41) Gemcitabine + cisplatin + bevacizumab (*n* = 34)	CA19-9
An [38]	2009	61	LAPC + metastatic	Gemcitabine Gemcitabine + oxaliplatin Gemcitabine + 5FU/CF	CA19-9
Koom [37]	2009	69	Borderline resectable + LAPC	Gemcitabine + radiation Paclitaxel + radiation	CA19-9
Yoo [39]	2011	84	LAPC	Radiation + 5-FU (*n* = 53) Radiation + capecitabine (*n* = 31)	CA19-9
Tsutsumi [46]	2012	90	LAPC + metastatic	Gemcitabine	CA19-9 SPAN-1
Boeck [36]	2013	68	LAPC + metastatic + recurrence	Gemcitabine Gemcitabine + erlotinib Gemcitabine + everolimus Gemcitabine + axitinib Gemcitabine + WX-671 Capecitabine Capecitabine + erlotinib Nab-paclitaxel	CA19-9 CEA
Azzariti [47]	2016	27	Metastatic	FOLFIRINOX (*n* = 21) Gemcitabine + nab-paclitaxel (*n* = 6)	CA19-9
Chiorean [45]	2016	454	Metastatic	Gemcitabine (*n =* 202) Gemcitabine + nab-paclitaxel (*n* = 252)	CA19-9
Robert [48]	2017	160	Metastatic	FOLFIRINOX (*n* = 85) Gemcitabine (*n* = 75)	CA19-9

LAPC = locally advanced pancreatic cancer, CA19-9 = carbohydrate antigen 19-9, CEA = carcinoembryonic antigen.

**Table 2:** Overview of studies including genetic markers as predictive biomarkers for chemotherapy response in pancreatic cancer patients.

Author	Year of Publication	Number of Patients	Stage of Disease	Treatment	Biomarker
Dong [54]	2009	131	Resectable	Gemcitabine + radiation Gemcitabine + cisplatin + radiation	SNPs (*MSH2* G322D, *MSH2* IVS12-6T>C, *MSH3* P231P, *TREX1* Ex14-460C>T, *TP73* Ex2+4G>A)
Tanaka [56]	2010	149	LAPC	Gemcitabine (+ radiation) Gemcitabine + cisplatin (+ radiation) Gemcitabine + oxaliplatin (+ radiation)	SNPs (*CDA* A-76C, *hENT1* A-201G)
Tjensvoll [64]	2016	14	LAPC (*n* = 2) + metastatic (*n* = 12)	Gemcitabine (*n* = 6) FOLFIRINOX (*n* = 8)	ctDNA (*KRAS*)
Cheng [61]	2017	13	Metastatic	Gemcitabine + nab-paclitaxel	ctDNA (*BRCA2*, *KRAS* 12G, *KRAS* G12V, *KRAS* G12D, *ERBB2*, *EGFR*, *KDR*)
Del Re [63]	2017	27	LAPC (*n* = 4) + metastatic (*n* = 23)	FOLFIRINOX (*n* = 13) Gemcitabine + nab-paclitaxel (*n* = 14)	ctDNA (*KRAS*)
Wang [67]	2017	62	LAPC (*n* = 21) + metastatic (*n* = 41)	Gemcitabine + cisplatin (*n* = 24) Gemcitabine + nab-paclitaxel (*n* = 14) Gemcitabine + oxaliplatin (*n* = 24)	lncRNAs (*PVT1, HOTTIP, MALAT1*)
Perets [62]	2018	17	Metastatic	Unknown	ctDNA (*KRAS*)

LAPC = locally advanced pancreatic cancer, ctDNA = circulating tumor DNA, SNP = single nucleotide polymorphism.

**Table 3:** Overview of studies including immunologic markers as predictive biomarkers for chemotherapy response in pancreatic cancer patients.

Author	Year of Publication	Number of Patients	Stage of Disease	Treatment	Biomarker
Palagani [84]	2012	4	Metastatic	5-FU + oxaliplatin + irinotecan and/or avastin	CD44+ cells
Vizio [78]	2012	58	LAPC + metastatic	Gemcitabine (*n* = 28) Gemcitabine + oxaliplatin (*n* = 23) Bevacizumab + capecitabine + radiation (*n* = 6) 5-FU + levofolinate calcium (*n* = 1)	IL-23 IL-17a TGF-β1
Wittwer [89]	2013	68	LAPC + metastatic + recurrence	Gemcitabine Gemcitabine + erlotinib Gemcitabine + everolimus Gemcitabine + axitinib Capecitabine Capecitabine + erlotinib Nab-paclitaxel	sRAGE
Wittwer [88]	2013	68	LAPC (*n* = 9) + metastatic (*n* = 42) + recurrence (*n* = 17)	Gemcitabine Capecitabine	sRAGE
Azzariti [47]	2016	27	Metastatic	FOLFIRINOX (21) Gemcitabine + nab-paclitaxel (6)	sCD40L
Gao [75]	2017	122	LAPC + metastatic	Gemcitabine (*n* = 119) Fluorouracil (*n* = 1) Capecitabine (*n* = 1) S1 (*n* = 1)	NLR
Usul Afsar [79]	2017	20	Metastatic	Gemcitabine (*n* = 11) Gemcitabine + platinum (*n* = 6) Gemcitabine + capecitabine (*n* = 3)	IL-18

5-FU = fluorouracil, LAPC = locally advanced pancreatic cancer, IL = interleukin, sRAGE = soluble receptor for advanced glycation end products, NLR = neutrophil-to-lymphocyte ratio, IL = interleukin, S1 = tegafur, gimeracil and oteracil potassium.

**Table 4:** Overview of PDAC clinical trials and the biomarkers to be investigated, registered at (www.clinicaltrials.gov).

Sponsor	ClinicalTrials.gov Identifier	Number of Patients	Stage of Disease	Treatment	Biomarker
**Circulating biomarkers**					
Asan Medical Center	NCT02749136	44	Borderline resectable, neoadjuvant treatment	FOLFIRINOX	Not mentioned
Massachusetts General Hospital	NCT01591733	48	Borderline resectable + resectable, neoadjuvant treatment	FOLFIRINOX + radiation	SNPs
UNC Lineberger Comprehensive Cancer Center	NCT01688336	45	Borderline unresectable + LAPC	FOLFIRINOX	CA19-9, CEA
Pancreatic Cancer Research Team	NCT01488552	60	Metastatic	Gemcitabine + nab-paclitaxel, followed by FOLFIRINOX	Miscellaneous, including CA19-9
Centre Georges Francois Leclerc	NCT03599154	100	Metastatic	Gemcitabine (+ nab-paclitaxel) FOLFIRINOX	RNA gene expression (Gemcitest)
Helse Stavanger HF	NCT02707159	70	Metastatic	Gemcitabine + nab-paclitaxel	Circulating tumor cells
National Cancer Center, Korea	NCT01333124	64	Resectable, neo-adjuvant treatment	Gemcitabine + radiation	Not mentioned
**Tissue biomarkers**					
Seoul National University Hospital	NCT02486497	40	Resectable, adjuvant treatment	Gemcitabine 5-FU	hENT1 protein expression
AHS Cancer Control Alberta	NCT01411072	20	Resectable, adjuvant treatment	Gemcitabine 5-FU	hENT1 protein expression
Grupo Hospital de Madrid	NCT01394120	60	Metastatic	FOLFIRINOX FOLFOX FOLFIRI Gemcitabine + capecitabine Gemcitabine Gemcitabine + erlotinib	Thymidilate Synthase, Thimidine Phosphorylase, ERCC1 and Topoisomerase I expression
Emory University	NCT01188109	25	Resectable, adjuvant treatment	Gemcitabine + cisplatin	ERCC1 gene expression

SNP = single nucleotide polymorphism, LAPC = locally advanced pancreatic cancer, CA19-9 = carbohydrate antigen 19-9, CEA = carcinoembryonic antigen, 5-FU = fluorouracil, hENT1 = human equilibrative nucleoside transporter 1, ERCC1 = excision repair 1, endonuclease non-catalytic subunit.
